# Microbiota–immune–enteric nervous system interactions in functional constipation: a narrative review and hypothesis-generating framework

**DOI:** 10.3389/fimmu.2026.1851825

**Published:** 2026-07-02

**Authors:** Dingzuo Ge, Yu Zhan, Yong Wen, Rong Wu, Qingxia Xu, Zhimin Ao, Yixin Shu, Xuegui Tang

**Affiliations:** 1 Hospital of Chengdu University of Traditional Chinese Medicine, Chengdu, China; 2 Affiliated Hospital of Integrative Chinese Medicine and Western Medicine of Chengdu University of Traditional Chinese Medicine (TCM), Chengdu, China

**Keywords:** enteric nervous system, functional constipation, gut microbiota, neuroimmune interactions, slow-transit constipation, systems biology

## Abstract

Functional constipation (FC), particularly slow-transit constipation (STC), is a heterogeneous disorder of gut–brain interaction that responds poorly to conventional therapies. Accumulating evidence links the microbiota, mucosal immunity, and the enteric nervous system (ENS); their mechanistic integration remains incomplete. In this narrative review, we propose a Trigger–Gateway–Hub–Effector framework as a heuristic and hypothesis-generating model to organize fragmented evidence on microbial-to-immune–neural interactions. Within this framework, dysbiosis-associated microbial metabolites, including short-chain fatty acids, bile acids, methane-related pathways, and lipopolysaccharide, are considered potential upstream “Triggers” that may modulate epithelial and immune homeostasis. “Gateway” processes refer to epithelial barrier vulnerability and mucosal immune changes that may permit microbial or inflammatory signals to affect deeper intestinal compartments. At the “Hub” level, interactions among muscularis macrophages, mast cells, enteric glia cells, and neurons are proposed to integrate these signals and contribute to ENS-adjacent neuroimmune stress. These processes may converge on downstream “Effector” alterations, including neuronal vulnerability, maladaptive plasticity, and disruption of the interstitial cells of Cajal network, particularly in severe or refractory STC. However, there is currently limited direct evidence to support a continuous causal chain linking microbiome-derived signals to dysfunction of the enteroneural system. Many of the proposed mechanisms are inferred from preclinical studies or related gastrointestinal disorders. Therefore, this framework should be interpreted as a testable conceptual model rather than a confirmed pathogenic sequence. We further discuss the translational implications from a systems biology perspective, emphasizing evidence-weighted therapeutic interpretation, mechanism-guided stratification, and integrated microbial-immune-ENS assessment. Future human-centered studies combining multi-omic profiling, spatial tissue analysis, and objective neuromuscular readouts are needed to refine this model and inform precision-oriented therapeutic strategies for FC/STC.

## 1 Introduction

Functional constipation (FC)is a common disorder of gut–brain interaction (DGBI). It has different clinical features in different patients. It also places a heavy burden on healthcare systems and society worldwide (1). Clinically, FC mainly includes infrequent bowel movements, excessive straining, hard stools and a lasting feeling of incomplete evacuation. These symptoms are common in clinical practice, with epidemiological studies reporting a global adult prevalence of approximately 10–15%, while pooled data from Chinese populations indicate a prevalence of about 8.5% (2, 3). FC can also be linked to psychological problems and a lower health-related quality of life. In some studies, this effect is close to that seen in chronic diseases such as rheumatoid arthritis or diabetes (4).FC is commonly classified into normal-transit constipation (NTC), slow-transit constipation (STC), and defecatory disorders (DD) according to colonic transit and anorectal function (5). Among these subtypes, STC is often considered a more difficult clinical phenotype because it is marked by delayed colonic transit and a weaker response to conventional drug therapies (6). But FC is not one single biological condition. Mechanisms found in STC, especially severe or refractory STC, should not be applied to all FC subtypes. This difference is important because much tissue-level evidence on enteric neuronal changes, interstitial cells of Cajal (ICCs), and immune remodeling comes from selected STC patients, not from the wider FC population.

Although FC is common, current treatments mainly focus on alleviating symptoms, and some patients still experience poor long-term outcomes. These treatments include osmotic and stimulant laxatives, secretagogues, and 5-HT4 receptor agonists. While these treatments can improve stool consistency or colonic motility, they do not directly target the early mechanisms of the disease (5). Real-world data indicate that many patients only experience partial or short-term relief of symptom. Some patients develop tolerance, and some remain dissatisfied with the results of their treatment. Many patients report that the benefits are insufficient or short-lived (7).

In the past, FC was mainly explained by abnormal motility. Its symptoms were often linked to poor neuromuscular coordination, delayed colonic transit, or pelvic floor dysfunction (6). This view explains several key clinical features, but it does not fully clarify why neuromuscular abnormalities develop or persist when obvious structural pathology is absent. It also cannot adequately explain why therapies aimed only at motor function show variable efficacy among patients.

Recent models of functional gastrointestinal disorders look beyond motility. They include interactions among gut microbiota, mucosal immunity, epithelial barrier function and neural circuits at the gut interface (8). The intestine is a dynamic ecosystem. Host tissues and gut microbiota send signals to each other continuously (9). Clinical and experimental studies report that patients with FC may altered gut microbial composition, including reduced relative abundance of taxa such as *Bifidobacterium* and *Lactobacillus*. But the role of these microbial changes in neuromuscular dysfunction is still unclear (10). This gap suggests that intermediate processes may link luminal microbial signals to changes in intestinal immune status and neuromuscular function (11).

Accumulating evidence supports a role for low-grade mucosal immune activation in modulating gut sensorimotor function, particularly in functional bowel disorder cohorts overlapping with constipation-predominant irritable bowel syndrome (IBS-C) (12). Unlike the pronounced inflammation characteristic of inflammatory bowel disease (IBD), functional bowel disorders may involve subtler changes in mucosal immune cell profiles, including alterations in mast cell density, distribution, or proximity to nerve fibers. Anatomical and histological studies describe close spatial apposition of immune cells, including mast cells (MCs), to enteric nerve fibers, providing a structural basis for immune–neural crosstalk (13). However, the extent to which these observations apply specifically to FC or STC remains incompletely established.

Within this context, dysbiosis-associated microbial signals may interact with epithelial barrier function and mucosal immune responses, thereby influencing enteric nervous system (ENS) activity in selected patient subgroups. However, direct evidence supporting a continuous microbiota–barrier–immune–ENS pathway in human FC/STC remains limited (9). Experimental studies and limited human tissue data suggest that ENScomponents or interstitial cells of Cajal (ICCs) networks may be influenced by environmental or immune factors, but these observations are preliminary, context-dependent, and most relevant to severe or refractory STC rather than FC as a whole (14). Importantly, this interpretation does not imply uniform or progressive neural injury across the FC population.

**Figure 1:**
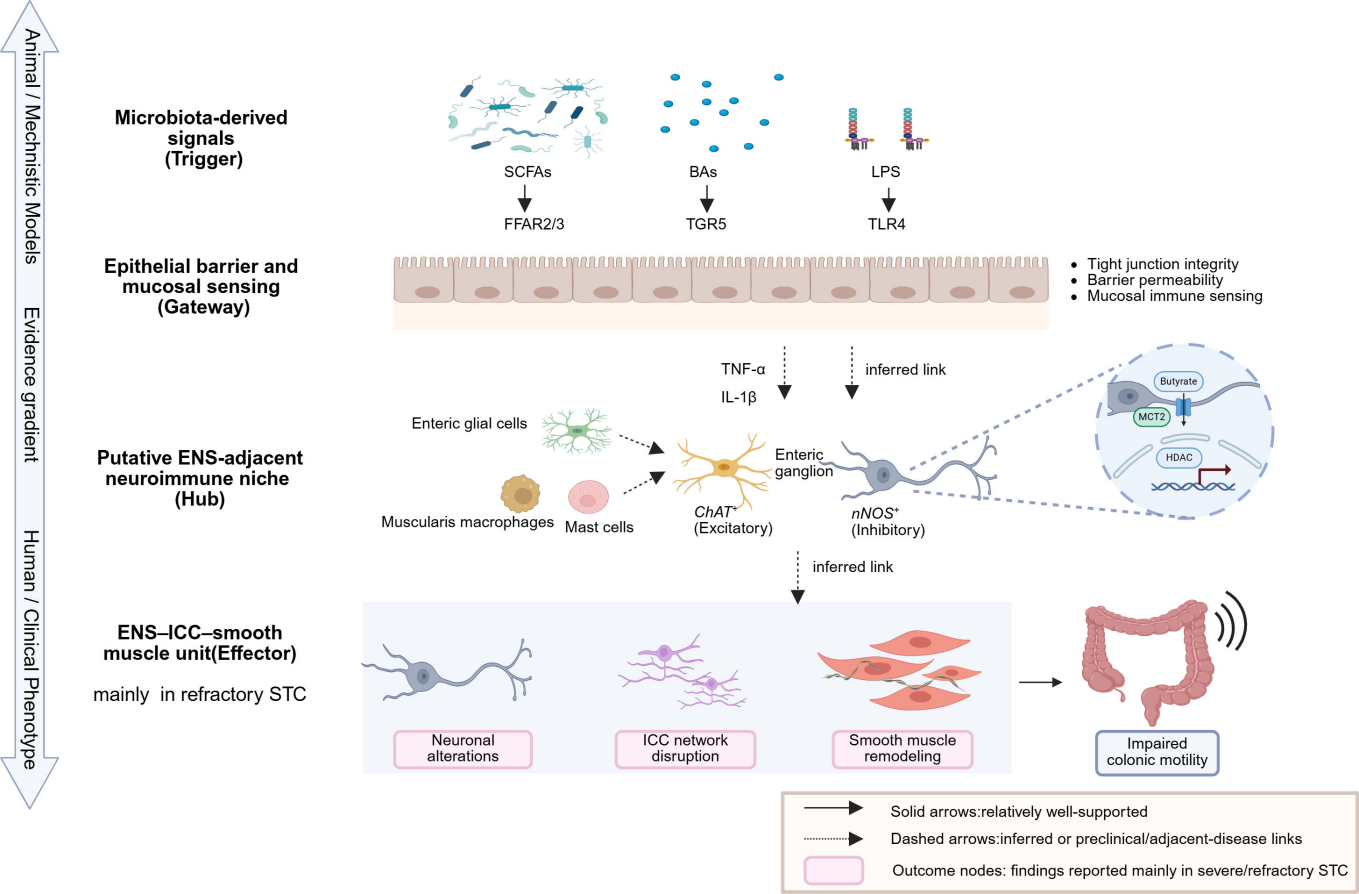
Trigger–Gateway–Hub–Effector framework for microbiota–immune–ENS interactions in FC/STC. The diagram summarizes the proposed Trigger–Gateway–Hub–Effector framework as a heuristic model rather than a confirmed linear pathogenic cascade. At the Trigger level, microbiota-derived signals, including short-chain fatty acids, bile acids, and lipopolysaccharide, may interact with epithelial and immune sensing pathways such as FFAR2/3, TGR5, and TLR4. At the Gateway level, epithelial barrier vulnerability and mucosal immune sensing are proposed to modulate host exposure to luminal microbial signals. The Gateway-to-Hub transition, involving possible communication between mucosal immune compartments and ENS-adjacent neuroimmune niches, remains largely inferred from preclinical and adjacent-disease evidence. At the Hub level, mast cells, muscularis macrophages, enteric glial cells, and neurons are shown as putative components of an ENS-adjacent neuroimmune niche. At the Effector level, neuronal alterations, ICC network disruption, and smooth muscle remodeling are presented as parallel downstream outcome nodes reported mainly in severe or refractory STC. Solid arrows indicate relatively well-supported biological or clinical associations, whereas dashed arrows indicate inferred links supported mainly by preclinical models, *in vitro* studies, or adjacent gastrointestinal disorders. Created in BioRender. Ge, D. (2026) https://BioRender.com/o06ua35.

In light of these observations, FC—particularly STC-predominant or refractory phenotypes—may be considered within a broader biological framework that extends beyond isolated motility impairment. From a mechanistic perspective, selected constipation phenotypes may involve microbiota-associated and immune-modulated alterations in enteric neural regulation, manifesting as functional neuromuscular dysregulation or, in more severe cases, tissue-level neuromuscular remodeling (15). To bring together different findings from microbiology, immunology, and neurogastroenterology, we propose a simple hypothesis-generating framework called the “Trigger–Gateway–Hub–Effector” model (**Figure 1**). This framework aims to summarize indirect and partly related evidence, identify mechanistic links that can be tested, and show current knowledge gaps. It should not be seen as a confirmed linear disease process.

The “Trigger” part focuses on changes in chemical signals from gut microbiota, not only changes in microbial composition. These signals include metabolites that may affect the epithelial, immune system, or nerves, such as short-chain fatty acids (SCFAs), bile acids, and indole derivatives. They also include increased exposure to r immune-stimulating microbial components such as lipopolysaccharides (LPS) (16, 17). Evidence for these factors in FC/STC is mainly associative, and their causal role in constipation-related neuromuscular dysfunction remains to be determined.

The “Gateway” part refers to epithelial barrier function and mucosal immune interfaces. Through this part, microbial signals in the gut lumen may be sensed, filtered, or increased. The “Hub” part refers to possible ENS-adjacent immune–glial–neuronal niche. It includes mast cells (MCs), muscularis macrophages, enteric glial cells (EGCs), and enteric neurons. Together, these parts connect luminal changes with local neuromodulatory responses (18–20). But evidence for Gateway-to-Hub signaling mainly comes from experimental models and related DGBIs., Direct evidence in FC or STC is still limited. Therefore, extrapolating of these mechanisms to constipation phenotypes requires caution (12, 14).

The “Effector” component reflects downstream neuromuscular alterations that may be associated with sustained immune–neural signaling. Immune-derived mediators including histamine, TNF-α, and oxidative stress-related factors, may contribute to functional changes in ENS signaling and, in selected severe cases, structural vulnerability within enteric neuromuscular networks (21). Based primarily on experimental studies and limited human tissue data, such processes have been proposed to relate to reduced excitatory cholinergic signaling, ICC network alterations, and impaired colonic motility in subsets of patients, particularly those with refractory STC (22). Whether these changes act as drivers, amplifiers, or consequences of prolonged stasis remains unresolved.

Understanding the microbiota–immune–ENS interations may help explain why conventional single-target therapeutic strategies in insufficient for some patients with FC/STC. However, therapeutic implications should be interpreted with evidence weighting. Some interventions, such as microbiota-directed strategies, have human symptom-based data, whereas immune-, glial-, or neuroprotective approaches remain supported mainly by preclinical or conceptual evidence. From a systems biology perspective, multi-layered interventions may be discussed as potential research directions, but they should not be presented as validated mechanism-based therapies without direct clinical and mechanistic confirmation. Accordingly, this narrative review aims to examine the “Trigger–Gateway–Hub–Effector” framework as a conceptual model for understanding microbiota–immune–ENS interactions in FC, with particular relevance to STC-predominant and refractory phenotypes. Specifically, we seek to: (1) summarize current evidence on how microbial derived metabolic signals may influence the intestinal epithelial and immune microenvironment; (2) characterize putative immune Gateway and ENS-adjacent Hub processes, with attention to MCs, macrophages, EGCs, and neurons(3) delineate proposed pathways associated with structural and functional alterations of the ENS and ICC network; and (4) discuss therapeutic implications using an evidence-weighted approach. By synthesizing evidence from microbiology, immunology, and neurogastroenterology, this review provides a working framework to guide future human-centered mechanism studies and precision-oriented strategies for FC/STC management.

## 2 Methods

This article is a narrative review rather than a systematic review or meta-analysis. Relevant literature was identified through searches of PubMed and Web of Science, supplemented by manual screening of reference lists from selected articles. Search terms included “functional constipation,” “slow-transit constipation,” “gut microbiota,” “immune activation,” “enteric nervous system,” “interstitial cells of Cajal,” “macrophage,” “mast cell,” and “intestinal barrier.” Human FC/STC studies were given priority. Mechanistic animal studies, *in vitro* studies, and studies from related gastrointestinal disorders were used when direct FC/STC evidence was not available. Because of the narrative nature of this review, no formal meta-analysis, risk-of-bias assessment, or systematic evidence grading was performed.

## 3 The Trigger: Dysbiosis-Derived Signals Shaping Immune–ENS Homeostasis

In the proposed Trigger–Gateway–Hub–Effector framework, changes in the gut microbiota are considered to be upstream modulators rather than deterministic drivers of FC/STC. In addition to changes in microbial composition, dysbiosis may alter the molecular signaling at the host-microbiome interface. These changes may impact epithelial barrier function, mucosal immune status, and enteric neuromuscular regulation. Current human evidence primarily demonstrates associations or potential modulatory effects. Mechanistic evidence primarily originates from animal, ex vivo and *in vitro* studies. from animal, ex vivo, and *in vitro* studies.

In this context, dysbiosis in FC/STC may include two changes. One is reduced metabolites that support epithelial and neuroimmune balance. The other is increased exposure to microbial signals that may affect mucosal immune responses. These signals may not directly determine colonic transit. Instead, they may create a permissive biological environment that increases barrier vulnerability and lowers the threshold for later immune–ENS perturbations. This section therefore interprets microbiota-derived signals as possible contributors to disease heterogeneity, rather than as established initiating causes of constipation.

### 3.1 Dysbiosis patterns that may affect ENS vulnerability

Many studies using 16S rRNA sequencing and metagenomic methods have reported changes in gut microbial composition in patients with FC compared with healthy controls. But the direction, size, and repeatability of these changes are very different across study groups (8). This difference may be related to study design, diet, medication use, constipation subtype, stool transit time, sampling method, and analysis method. So, a clear microbial signature for FC/STC has not yet been found.

Several patterns that may have functional meaning have been reported (23). These include lower levels of butyrate-producing taxa within the Ruminococcaceae and Lachnospiraceae families, such as *Faecalibacterium* and *Roseburia*. These bacteria help produce short-chain fatty acids (SCFAs) and support epithelial metabolism (24, 25). At the same time, higher levels of methanogenic archaea, particularly *Methanobrevibacter smithii*, has been linked to higher breath methane levels and delayed intestinal transit. This suggests a possible link between methane production and constipation-related motility phenotypes (26, 27). Other changes, such as changes in Enterobacteriaceae or mucin-degrading *Akkermansia* have also been reported in some FC cohorts; But their specific roles in mucosal barrier stress, mucosal immune activation, or immune–ENS interactions are still unclear (8, 28).

### 3.2 Potential loss of neuro-protective metabolic support

The ENS responds to many metabolites from gut microbes. Experimental studies suggest that changes in these metabolic signals can influence ENS development, maintenance, and function (29, 30). In FC/STC, altered metabolite availability may be one possible way that dysbiosis affects epithelial, immune, and neuromuscular balance. This may happen without clear neuronal damage. But this idea has not been fully confirmed in human constipation. It should be seen as a possible modulatory mechanism, not a confirmed disease process.

#### 3.2.1 Short-chain fatty acids

Microbial fermentation of dietary fiber produces SCFAs, primarily acetate, propionate, and butyrate. In STC and related functional bowel disorders, lower levels of SCFA-producing bacteria has been reported. Some studies have also found lower fecal or luminal SCFA concentrations (31). Under physiological conditions, SCFAs support epithelial energy metabolism, maintain barrier integrity, and help regulate host gene expression partly by inhibiting histone deacetylase (HDAC). In this way, they help maintain cellular homeostasis and mucosal resilience (32). SCFAs can also activate free fatty acid receptors, such as FFAR2 and FFAR3, on enteroendocrine cells, enteric neurons, and enterochromaffin cells. This links microbial metabolism with serotonin signaling and motility regulation (33). In experimental systems, SCFA-induced activation of these receptors has been associated with serotonin (5-HT) synthesis through increased tryptophan hydroxylase-1 (TPH1) expression (34, 35). 5-HT is important for intrinsic primary afferent neuron activation and the start of the peristaltic reflex. But it is still unclear how much this SCFA-related pathway works in human FC/STC (36, 37). In STC, gut microbiota dysbiosis is often linked to lower SCFA levels. This reduction may weaken epithelial and neuroimmune homeostatic support. It may also affect TPH1 expression and lower colonic 5-HT availability. These changes may contribute to colonic hypomotility in some patients (38, 39). But these links are mainly associative in human studies. In animal models, butyrate supplementation has been reported to alter the balance between excitatory *ChAT*-positive and inhibitory *nNOS*-positive myenteric neurons. It has also been reported to improve colonic motility (40). Similar neuronal subtype changes have not been shown consistently in human FC or STC tissues. So, reduced SCFA availability in constipation is better seen as a potential loss of trophic and signaling support not as a direct cause of ENS dysfunction.

#### 3.2.2 Bile acid signaling

In addition to the classic role in dietary fat emulsification, bile acids act as signaling molecules, regulating gastrointestinal motility, secretion, and mucosal barrier function of the digestive tract (41). Primary bile acids are synthesized by cholesterol in hepatocytes and binds to taurine or glycine. In the colon, the process of decomposition and 7α-dehydroxylation is initiated by the gut microbiota, generating the production of secondary bile acids, such as deoxycholic acid (DCA) and lithocholic acid (LCA) (42, 43).It has been established that secondary bile acids, particularly DCA and LCA serve as endogenous ligands for G protein-coupled bile acid receptor TGR5/GPBAR1 (44). In STC, alterations in fecal bile acid composition have been documented, including shifts in the relative proportion of primary and secondary bile acids (45). Furthermore, a decline in TGR5 expression has been found in colonic mucosal biopsies from patients with STC (46). TGR5 is expressed in several cell types involved in motility regulation, including enterochromaffin (EC) cells and both excitatory cholinergic and inhibitory nitrergic neurons in the myenteric plexus (47). Activation of TGR5 on EC cells stimulates 5-HT release, whereas activation on IPANs may induce calcitonin gene-related peptide (CGRP) release (48, 49). These mediators participate in peristaltic reflex pathways that support luminal propulsion (50).Studies in *Tgr5-deficient (Tgr5^-^/^-^)* mice show slower whole-gut and colonic transit, reduced defecation frequency, and decreased fecal water content, resembling features of STC (49). Human pharmacogenetic evidence also supports the relevance of this pathway. A single nucleotide polymorphism (SNP) in the TGR5 gene, rs11554825, has been associated with altered colonic transit time in patients with functional gastrointestinal disorders (51). These findings suggest that bile acid–TGR5 signaling may modulate intestinal transit, although its direct mechanistic contribution to human STC remains incompletely established.

Emerging evidence suggests that fecal microbiota transplantation (FMT) may alleviate constipation symptoms partly by reshaping gut microbiota composition, altering fecal bile acid profiles, and influencing TGR5-mediated signaling (52). However, these mechanisms are supported mainly by animal, ex vivo, or indirect human evidence, Their direct contribution to impaired motility in human STC is likely context-dependent and may be influenced by fecal bile acid composition, gut microbiota configuration, receptor expression, diet, and baseline transit phenotype.

### 3.3 Low-grade pro-inflammatory biasing of the mucosal immune niche

Alterations in gut microbiota composition in functional gastrointestinal disorders, including FC, have been associated with markers of low-grade immune activation and subtle mucosal immune changes (53, 54). Rather than reflecting overt inflammatory pathology, these changes may indicate a shift in immune tone that influences epithelial and neuroimmune signaling at the gut interface. However, evidence directly linking dysbiosis-induced immune activation to ENS dysfunction in human FC/STC remains limited.

#### 3.3.1 Lipopolysaccharide and TLR4 signaling

Diet-induced dysbiosis, particularly under high-fat feeding conditions, has been associated with increased luminal and circulating LPS, a component of Gram-negative bacterial membranes (55). Low-grade LPS translocation, sometimes referred to as metabolic endotoxemia, can active Toll-like receptor 4 (TLR4) signaling in immune and non-immune cells. Although TLR4 expression has been documented in enteric neural networks, direct evidence that circulating or luminal LPS activates enteric neurons or glial cells in humans FC/STC remains limited (56).

Mice harboring a spontaneous mutation in the TLR4 gene, rendering them hyporesponsive to LPS, exhibit delayed intestinal transit, reduced stool frequency, and a fewer myenteric neurons (57). Bone marrow transplantation experiments suggest that this effect is mediated by TLR4 expression on non-hematopoietic cells, including neurons and glia, supporting a potential neurotrophic role for microbial sensing (57). In murine models, chronic dysbiosis associated with elevated LPS exposure has been linked to TLR4-dependent loss of enteric nitrergic neurons and delayed colonic transit (58, 59).

Under conditions of chronic dysbiosis or inflammation, increased intestinal permeability may permit bacterial products such as lipopolysaccharide to interact with resident innate immune cells, including muscularis macrophages. Activation of TLR4–MyD88–NF-κB signaling may promote production of pro-inflammatory cytokines, including tumor necrosis factor-alpha (TNF-α), interleukin-1 beta (IL-1β), and IL-6 (60, 61). In models of chronic intestinal inflammation, sustained activation of this pathway has been associated with enteric neuronal injury and ICC network disruption, features that overlap with observations reported in severe STC cohorts (57, 62).

These findings suggest that sustained innate immune sensing may modulate ENS integrity in experimental settings. However, whether analogous TLR4-dependent mechanisms operate in human FC or STC remains unresolved. Current evidence supports a context-dependent modulatory or amplifying role rather than a primary pathogenic driver (63).

#### 3.3.2 Tryptophan metabolism bias

Tryptophan metabolism represents a key regulatory node in microbiota–host interactions (64). Dietary tryptophan can be metabolized through host serotonin synthesis, the kynurenine pathway, or microbial indole pathways, with gut microbes modulating flux distribution among these routes (65). Microbial indole derivatives activate the aryl hydrocarbon receptor (AhR) and contribute to epithelial barrier maintenance and immune regulation. Alterations in tryptophan metabolism and indole profiles has been associated with immune dysregulation in several gastrointestinal disorders (66, 67).

Tryptamine, produced through bacterial decarboxylation, acts as a physiological ligand for the 5-HT4 receptor (5-HT4R), which is expressed in the colonic epithelium (68). Unlike neuronal 5-HT4R, which is directly involved in motility pathways, epithelial 5-HT4R receptor activation by tryptamine increases ionic flux and anion-dependent fluid secretion (69). This suggests that microbial tryptamine may influence bowel function through epithelial secretory mechanisms as well as indirect effects on luminal hydration.

Indole, the most abundant microbial tryptophan metabolite, influences gut homeostasis through aryl hydrocarbon receptor (AhR) signaling via its derivatives and can directly activate transient receptor potential ankyrin 1 (TRPA1) channels (16, 70). While AhR activation by indole derivatives supports barrier integrity and mucosal immune regulation, indole-induced glucagon-like peptide-1 (GLP-1) secretion is primarily mediated through TRPA1 secretion on enteroendocrine L-cells (71). Indole triggers Ca²^+^ mobilization in L-cells can lead to rapid GLP-1 secretion. Because GLP-1 modulates gastrointestinal motility and satiation, dysregulated indole signaling may plausibly influence neuroendocrine unction in constipation-related phenotypes (72). However, this remains largely inferential in human FC/STC.

Diversion of tryptophan away from serotonin synthesis may reduce substrate availability for enterochromaffin cell–derived serotonin, thereby modulating motility-related signaling and influencing immune pathways through kynurenine and indole metabolites (73). Nonetheless, direct evidence linking altered tryptophan metabolism to constipation phenotypes in humans remains limited. This pathway should therefore be considered biologically plausible but incompletely validated.

### 3.4 Integrated perspective

Collectively, microbiota-derived signals associated with dysbiosis—including altered SCFA and bile acid profiles, increased LPS exposure, and shifts in tryptophan metabolism—should be regarded as potential modulators of neuroimmune regulation rather than direct determinants of colonic motility. By biasing epithelial mucosal immune tone and diminishing homeostatic support for the ENS, these signals may create a permissive environment that facilitates downstream barrier vulnerability and immune–neural interactions. This conceptual interpretation provides a bridge to subsequent sections on Gateway and Hub process while remaining consistent with current limitations in human evidence (74). A structured, evidence-weighted overview of representative microbiota-derived signals, their putative host targets, evidence sources, and interpretive strength is provided in **Table 1**.

**Table 1:** Evidence-weighted overview of representative microbiota-derived signals potentially involved in microbiota–immune–ENS interactions in FC/STC.

Microbial signal/pathways	Primary target cells/pathways	Potential relevance to ENS/colonic motility	Main evidence source	Interpretation strength
Short-Chain Fatty Acids (SCFAs)	Epithelial cells, EC cells, EECs, enteric neurons; FFAR2/3	Barrier support, 5-HT signaling, neuronal trophic support	Human associative data and animal/*in vitro* mechanistic studies	Potential modulator; not proven causal
Secondary Bile Acids	EC cells, myenteric neurons; TGR5	5-HT/CGRP release, peristaltic reflex, transit regulation	Human STC observations, Tgr5 mouse models, and genetic association studies	Plausible pathway; context-dependent
LPS/TLR4	Innate immune cells, macrophages, neurons, glia	Innate immune activation, cytokine release, ENS stress	Mainly animal and inflammatory models	Possible amplifier; not established driver
Tryptophan Metabolites	Epithelial cells, L cells, EC cells; AhR/TRPA1/5-HT4R	Barrier regulation, GLP-1 release, secretion, neuroendocrine signaling	Mainly preclinical and adjacent disease evidence	Hypothesis-generating
Methane metabolism	Luminal environment, motility-related physiology	Delayed transit and constipation phenotype	Human breath-test association studies	Useful stratification marker; mechanism incomplete
Immune-derived Mediators	MCs, macrophages, EGCs, ENS, ICCs	Neuronal excitability, glial activation, ICC disruption	Human refractory STC tissue studies and animal models	Histological relevance; causality unresolved

## 4 The gateway: barrier vulnerability and immune micro-environment remodeling

Within the proposed Trigger–Gateway–Hub–Effector framework, the intestinal epithelium is viewed as a potential “Gateway” linking luminal microbial signals to subepithelial immune compartments and, indirectly, to ENS-adjacent microenvironments. In this review, the Gateway does not refer to overt epithelial breakdown or inflammatory ulceration. Rather, it refers to subtle and context-dependent changes in epithelial permeability, mucus organization, tight-junction regulation, and mucosal immune sensing that may increase exposure of host tissues to microbial-derived signals.

Direct human evidence demonstrating a continuous pathway from epithelial barrier vulnerability to ENS dysfunction in FC/STC remains limited. Therefore, the mechanistic concepts discussed in this section are largely inferred from experimental models, IBS, IBD, metabolic disease, postoperative ileus, and other adjacent gastrointestinal contexts. These data support the biological plausibility of Gateway processes but should not be interpreted as direct proof of pathogenesis in human constipation.

### 4.1 Epithelial barrier integrity: structural preservation versus functional vulnerability

Epithelial barrier integrity reflects the capacity of the intestinal mucosa to permit selective absorption of nutrients and water while restricting excessive translocation of luminal antigens and microbial products. In FC and STC, available evidence does not indicate gross epithelial disruption. However, functional barrier vulnerability has been proposed, largely on the basis of IBS, IBD, and experimental studies–as a permissive factor that may facilitate microbiota–immune interactions at the mucosal interface.

Structurally, the epithelial barrier consists of a monolayer of enterocytes and specialized epithelial cells, including goblet and Paneth cells, interconnected by tight junctions (TJs), adherens junctions, and desmosomes (75). TJ complexes, comprising claudins, occludin, and zonula occludens proteins, maintain paracellular selectivity and are dynamically regulated by cytokines, microbial metabolites, and cellular stress. In experimental systems, disruption of TJ organization increases paracellular permeability and is associated with enhanced engagement of mucosal immune receptors and low-grade inflammatory signaling (76, 77).

Dysbiosis may further increase barrier susceptibility by reducing metabolites such as SCFAs, which support tight junction stability, mucus production, and epithelial energy metabolism. Limited and heterogeneous clinical observations in chronic constipation suggest reduced mucus thickness and increased epithelial–bacterial contact, which may be associated with mucosal immune responses (78). However, whether these findings represent a primary barrier defect, a consequence of altered transit, or a secondary adaptation to changes in the luminal environment remains uncertain.

Mechanistically, experimental systems indicate that increased epithelial permeability may facilitate the passage of microbial components into subepithelial compartments, where they can engage pattern-recognition receptors on epithelial, immune, stomal, or glial cells. Such signaling may influence mucosal immune tone and, under some conditions, affect communication with deeper neuromuscular compartments. However, direct human evidence linking epithelial barrier alterations to ENS structural or functional impairment in FC or STC is lacking. Most mechanistic insights are extrapolated from IBS, IBD, metabolic disease or experimental models.

Clinically, barrier vulnerability is therefore best interpreted as a permissive condition that may enable or amplify downstream immune–ENS interactions rather than as an independent driver of motility failure. Within the proposed framework, epithelial dysfunction represents a candidate Gateway process that may increase exposure of mucosal and submucosal immune compartments to luminal microbial signals, thereby setting the stage for potential communication with ENS-adjacent immune niches.

### 4.2 Translocation of microbial signals and metabolic endotoxemia

Translocation of microbial signals means that molecules from gut microbiota pass across the epithelial barrier into subepithelial or systemic area. In these areas, they may activate immune pathways and indirectly influence neuromuscular regulation. Metabolic endotoxemia is one example of this process. It has mainly been studied in experimental systems, obesity, and metabolic disease, with only conceptual relevance to FC/STC.

Under conditions of increased epithelial permeability, luminal components such as LPS may pass through paracellular or transcellular routes into the lamina propria or systemic circulation. LPS can engage pattern-recognition receptors, particularly Toll-like receptor 4 (TLR4), on innate immune cells. They can active MyD88-dependent signaling cascades and inducing pro-inflammatory cytokine production, including TNF-α and IL-6. In inflammatory and metabolic contexts, these mediators modulate neuroimmune communication and alter gastrointestinal motility (79, 80).

Although metabolic endotoxemia is most clearly documented in obesity and metabolic disease. But its main parts, including barrier susceptibility, microbial signal entry, and long-term low-grade immune activation, provide a useful conceptual framework for FC/STC. This framework may help explain how luminal microbial signals affect intestinal immune status. However, direct evidence demonstrating chronic LPS translocation or TLR4-driven immune activation in human FC or STC remains limited. Current data mainly support biological plausibility, not a confirmed disease mechanism.

Emerging conceptual models further suggest that structural heterogeneity of circulating LPS and context-dependent receptor engagement may shape immunomodulatory, immunomodulatory, ortolerogenic responses (81). These uncertainties highlight important knowledge gaps regarding the molecular identity, activation thresholds, spatial distribution, and cellular targets of translocated microbial signals within intestinal tissues.

A key unresolved question is whether microbial-derived signals that cross the mucosal barrier can influence immune microenvironments surrounding enteric ganglia in human FC/STC. Addressing this question will require spatially resolved human studies that connect epithelial barrier markers, microbial products or metabolites, immune-cell localization, and ENS-adjacent tissue changes within the same patients. This issue provides the conceptual basis for the next step in the framework: the formation or remodeling of periganglionic immune niches. Although not derived from constipation models, a colitis study reported macrophage-dependent disruption of the myenteric plexus barrier, suggesting that inflammatory remodeling can increase access of immune or microbial-associated signals to ENS-adjacent compartments. This finding supports the biological plausibility of a Gateway-to-Hub transition, but it should be interpreted as adjacent-disease mechanistic evidence rather than direct evidence for FC/STC pathogenesis (82).

### 4.3 Recruitment and remodeling of the periganglionic immune niche

Following epithelial barrier vulnerability, microbial-derived signals may contribute to immune-cell activation or recruitment within intestinal tissue compartments. In experimental systems, such processes can extend toward regions surrounding enteric ganglia, where immune cells interact with enteric neurons and enteric glial cells. In the present framework, this region is referred to as the periganglionic immune niche, meaning immune cells and stromal components located near enteric ganglia that may participate in localized ENS modulation. Direct evidence for this Gateway-to-Hub transition in human FC/STC remains limited.

Recent single-cell RNA sequencing data have provided evidence of immune-stromal remodeling in human STC. In particular, XCL2^+^ CD8^+^ T cells were reported to promote chronic inflammation by inducing pro-inflammatory cytokine secretion in fibroblasts through IFNG and TNFSF14-related pathways. This inflammatory environment was associated with oxidative stress and intestinal dysfunction in STC patients and the NECTIN2-TIGIT pathway was identified as a potential regulatory axis (83). These findings support the presence of disease-associated immune reprogramming in the colonic microenvironment of STC. However, they do not by themselves establish that barrier-derived microbial signals initiate ENS-adjacent immune remodeling.

Within the gastrointestinal muscularis, immune populations are spatially organized, with muscularis macrophages (MMs) representing a major resident population positioned in proximity to enteric neurons and glia. High-dimensional profiling indicates that macrophages constitute a principal immune subset in the muscularis, whereas monocytes, dendritic cells, innate lymphoid cells, and adaptive immune populations occupy adjacent but distinct microdomains (84). This spatial organization provides an anatomical for specialized immune–neural interfaces.

Immune recruitment and maintenance within the muscularis are influenced by chemokine and cytokine gradients generated by epithelial cells, enteric neurons, and EGCs, stromal cells, and resident immune populations. Colony-stimulating factor 1 (CSF1), produced by neurons and glia, supports MM survival and differentiation and may regulate monocyte recruitment within the muscularis compartment (20). In turn, MMs can secrete mediators such as bone morphogenetic protein 2 (BMP2), which signal back to ENS circuits and modulate gut motility in experimental models, consistent with bidirectional neuroimmune communication (85).

Functionally, periganglionic immune cells may adopt pro-inflammatory or tissue-remodeling phenotypes, releasing cytokines such as IL-1β and TNF-α that can sensitize adjacent neurons, influence synaptic signaling, or promote low-grade neuroimmune stress in experimental settings (86). In STC models, a shift from anti-inflammatory M2-like macrophages toward pro-inflammatory M1-like macrophage phenotypes has been associated with increased IL-6 and TNF-α levels and enteric neurons loss (87, 88). MMs normally provide neurotrophic support through BMP2 secretion (89); however, this supportive pathway may be altered under inflammatory or dysbiotic conditions and has been proposed to contribute to neuronal vulnerability in STC-related models (90).

Downstream tissue changes in severe or refractory STC may include loss of enteric neurons, enteric glial cells (EGCs), and ICCs, although the upstream immune mechanisms remain incompletely defined (91, 92). EGCs provide structural and metabolic support for neuronal networks and help maintain neurotransmitter balance in the synaptic microenvironment (93, 94). Their depletion, when observed together with neuronal or ICC alterations, may weaken neuro-enteric coordination and impair propulsive motor patterns (95, 96). Such tissue-level alterations may contribute to the refractory nature of advanced STC; however, whether they are drivers, amplifiers, or consequences of prolonged stasis remains unresolved (97).

In human FC/STC, direct evidence for immune-cell recruitment, phenotypic polarization, and ENS subtype-specific vulnerability remains limited, representing a critical gap in current knowledge. The human single-cell data described above provide important that disease-associated immune reprogramming can occur within the STC colonic microenvironment, but further studies are needed to determine whether this remodeling is causally linked to epithelial Gateway dysfunction, microbial translocation, or ENS impairment.

Within the proposed framework, the periganglionic immune niche serves as a putative Gateway-to-Hub transition zone, translating upstream barrier-associated and immune-derived signals into localized immune–ENS interactions. This conceptualization sets the stage for the next section, which focuses on discrete immune–neuronal effector domains, beginning with mast cell–nerve microdomains.

## 5 The hub: immune–glial–neuronal interactions in ENS vulnerability

**Figure 2:**
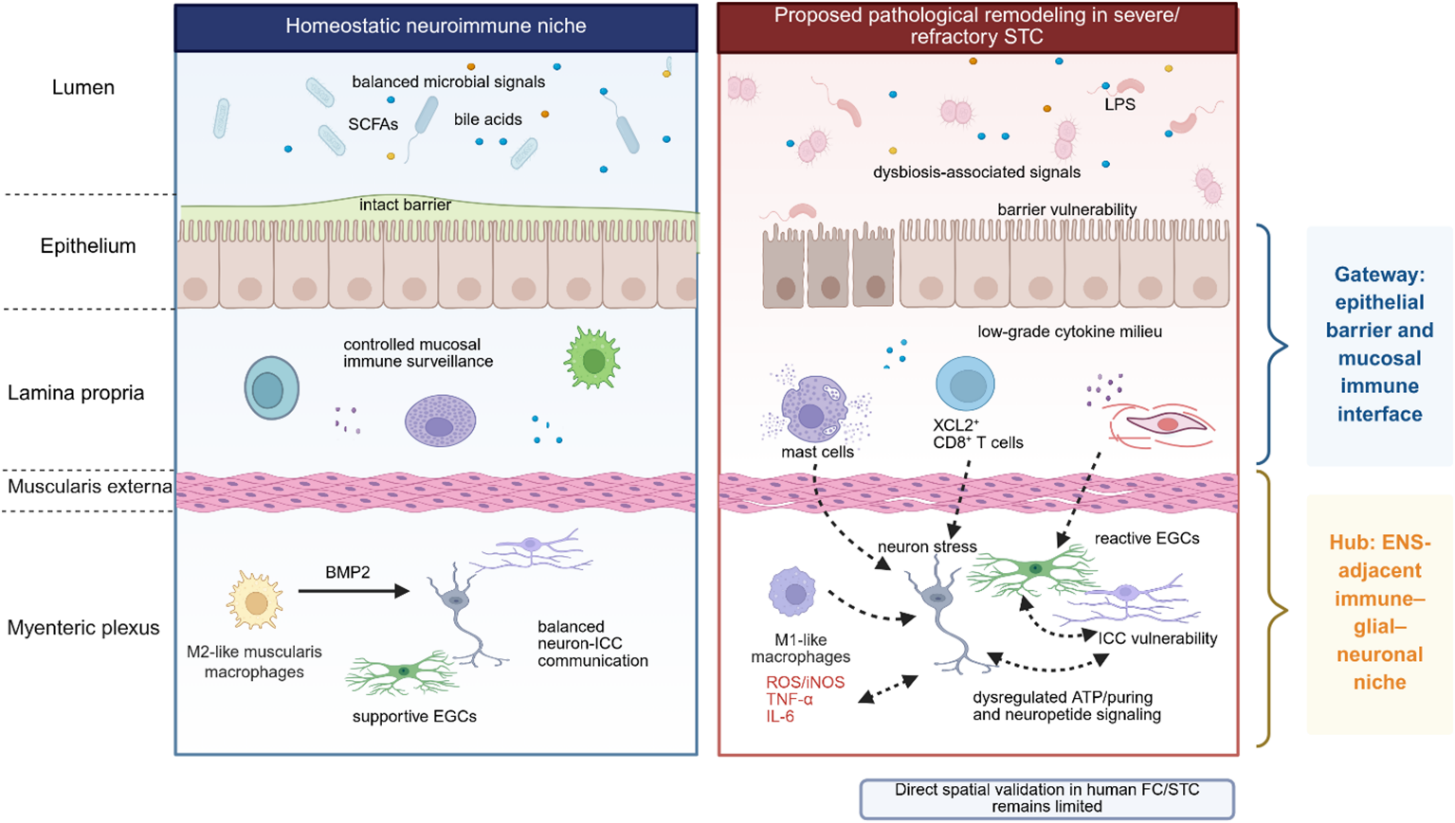
Anatomical organization of the proposed Gateway-to-Hub transition in FC/STC. The schematic illustrates homeostatic and proposed pathological neuroimmune organization across the intestinal wall. The Gateway is defined as the epithelial barrier and mucosal immune interface, including the epithelium and lamina propria, where luminal microbial signals may be sensed or filtered. The Hub is defined as the ENS-adjacent immune–glial–neuronal niche within the muscularis externa and myenteric plexus. Under homeostatic conditions, epithelial integrity, controlled mucosal immune surveillance, muscularis macrophage-derived BMP2 signaling, enteric glial support, and balanced neuron–ICC–smooth muscle communication contribute to neuromuscular homeostasis. In severe or refractory STC and related experimental contexts, barrier vulnerability, low-grade immune remodeling, mast cell activation, macrophage phenotypic shifts, reactive enteric glial signaling, and altered neuron–ICC communication are proposed to contribute to localized neuroimmune stress. Solid arrows indicate relatively well-supported physiological interactions, whereas dashed arrows indicate inferred or preclinical/adjacent-disease links. Direct spatial validation of this Gateway-to-Hub transition in human FC/STC remains limited. Created in BioRender. Ge, D. (2026) https://BioRender.com/ngjp2s7.

In the proposed Trigger–Gateway–Hub–Effector framework, the “Hub” denotes a putative ENS-adjacent microenvironment in which immune, glial, neuronal, stromal, and microbial-related signals may be locally integrated. This concept refers primarily to the muscularis externa and regions surrounding enteric ganglia, where immune cells, enteric glial cells (EGCs), and neurons are anatomically positioned to engage in bidirectional communication (**Figure 2**). However, the Hub should be interpreted as a conceptual integration of independently described cellular interactions rather than as a spatially or functionally validated unit in human FC/STC.

Current support for this integrated Hub comes mainly from experimental models, organoid systems, inflammatory conditions, and adjacent disorders of gut–brain interaction. Direct spatial validation in human constipation remains limited. Therefore, the following sections discuss mast cell–nerve microdomains, muscularis macrophage plasticity, and reactive enteric glia as biologically plausible components of an ENS-adjacent neuroimmune niche, while distinguishing established observations from inferred mechanisms.

Recent advances using optogenetics-enabled intestinal organoid systems provide proof-of-concept evidence that neuronal activity can be interrelated with microbial signals in localized epithelial-neural-immune contexts. In these models, frequency-specific activation of enteric neurons interacts with microbial signals, such as *T. ramosa*, and influences immune lineage specification and barrier-associated functions (98). Although these findings are derived from *in vitro* systems, they support the broader concept that neuronal activity and microbial cues can interact within defined gut microenvironments). Their relevance to chronic FC/STC remains to be directly tested.

Beyond innate immunity, emerging evidence also implicates adaptive immune pathways in microbiota-associated modulation of intestinal motility. In experimental models, T-cell signaling induced by defined microbial colonization has been shown to promote enteric neurogenesis through IL-1β- and IL-17A–associated pathways (99). These findings suggest that immune cells can influence ENS plasticity under specific experimental conditions. However, whether comparable adaptive immune–ENS programs operate in human FC/STC, and whether they contribute to delayed transit or represent compensatory responses, remains unresolved.

Rather than representing overt inflammation, the Hub is proposed to act as a signal-modulating interface that may transform low-intensity microbial or immune cues into spatially restricted neuroimmune stress. Within this framework, individual microbial metabolites or inflammatory mediators are unlikely to exert substantial neuronal effects in isolation. Instead, coordinated cellular interactions may cumulatively alter ENS resilience and contribute to functional impairment in selected FC/STC phenotypes without classical inflammatory pathology.

### 5.1 Mast cell–nerve microdomains

Mast cell–nerve microdomains refer to spatially restricted regions in which MCs are in close apposition to enteric or extrinsic nerve fibers, allowing rapid and bidirectional neuro–immune communication. These microdomains represent early transduction sites within the immune–ENS axis, where microbial-, immune, or stress-related stimuli are translated into localized changes in neuronal signaling. However, their direct contribution to impaired colonic motility in human FC/STC remains uncertain.

MCs are distributed across mucosal, submucosal, and muscularis layers and frequently localize near intrinsic and extrinsic nerve fibers. A substantial proportion of intestinal MCs reside near sensory and autonomic neurons, forming anatomically constrained units that facilitate efficient signal exchange (100). This spatial proximity provides a structural basis for mast cell–nerve communication but does not by itself establish disease causality in constipation.

Functionally, MCs express diverse receptor systems, including FcϵRI, Toll-like receptors, and G protein–coupled receptors, enabling responsiveness to immunoglobulin-mediated cues, microbial components, and neuropeptides (101). Upon activation, MCs release mediators such as histamine, tryptase, serotonin (5-HT), prostaglandins, and cytokines, which can modulate neuronal excitability and synaptic signaling through histamine, protease-activated, and serotonin receptors expressed on enteric neurons (102). Reciprocally, neurons secrete neuropeptides, including substance P, CGRP, and VIP, which can further activate MCs and establish localized feedback loops (103).

In functional gastrointestinal disorders, most notably irritable bowel syndrome, human studies have demonstrated increased mast cell density near enteric nerve fibers, correlating with visceral hypersensitivity and pain (104). Although FC/STC and IBS are overlapping but distinct conditions, these findings provide a conceptual analogy for spatially restricted immune–neural signaling. In constipation phenotypes, analogous mast cell–nerve interactions may influence ENS excitability, secretion, or local immune tone, but they should not be interpreted as direct evidence that mast cells drive motility failure.

At present, direct evidence linking MC-nerve microdomain activity to impaired motility in human FC/STC remains limited (104). Key unresolved issues include the dominant mediator profiles in constipation phenotypes, the extent to which microbial dysbiosis alters MC activation thresholds, and whether MC–nerve signaling increases ENS vulnerability or represents a secondary adaptive response (105).

### 5.2 Macrophage phenotypic plasticity

Macrophage phenotypic plasticity describes the capacity of intestinal macrophages, particularly those within the muscularis externa, to adopt distinct functional states in response to local environmental cues. Within the proposed ENS-adjacent Hub, muscularis macrophages may act as context-dependent modulators of ENS structure and function by integrating microbial, immune, stromal, and neuronal signals. This concept is strongly supported by experimental neurogastroenterology studies, but direct validation in human FC/STC remains incomplete.

Muscularis macrophages (MMs) reside in proximity to enteric neurons and represent a major immune population within the muscularis externa. Under steady-state conditions, these macrophages exhibit tissue-adaptive and anti-inflammatory properties that support homeostasis and participate in motility regulation (106).

Mechanistic studies demonstrate reciprocal signaling between enteric neurons and muscularis macrophages. Enteric neurons supply colony-stimulating factor 1 (CSF1), which is important for macrophage maintenance, whereas MMs secrete bone morphogenetic protein 2 (BMP2), which modulates neuronal activity through BMP receptor signaling (89). These bidirectional interactions illustrate how macrophage phenotypes can be shaped by neuronal cues and, in turn, influence ENS function.

Under inflammatory, infections, or stress-related conditions, macrophages can shift toward pro-inflammatory or tissue-remodeling states characterized by increased cytokine production, oxidative stress, and altered trophic signaling. In acute inflammatory models, including postoperative ileus, macrophage activation is associated with transient neuronal dysfunction, synaptic remodeling, and impaired motility (107). These findings should not be extrapolated as evidence of comparable inflammatory magnitude or neuronal injury in chronic FC/STC. Rather, they provide proof-of-principle that macrophage-dependent pathways can modulates ENS integrity and motility under defined conditions.

Enteric glial-derived signals may further shape macrophage phenotypes. During muscularis inflammation, glial activation can promote monocyte recruitment and macrophage differentiation through chemokines and growth factors, including CCL2 and CSF1 (108). In FC/STC, maladaptive macrophage transitions could plausibly contribute to persistent low-grade immune biasing and altered neuroimmune homeostasis, although direct human evidence remains scarce. Together with experimental evidence implicating T cells in e ENS plasticity (99), these observations support the concept that the muscularis microenvironment can act as a multicellular signaling interface. Whether this interface becomes chronically dysregulated in human constipation remains an important unresolved question.

### 5.3 Reactive enteric glial cells

Reactive EGCs refer to context-dependent changes in glial phenotype and signaling induced by environmental perturbations such as barrier dysfunction, immune activation, or injury. Rather than representing a uniform pathological state, enteric glial reactivity encompasses transcriptional, metabolic, calcium-signaling, and mediator-release adaptations that may reshape local neuroimmune interactions within ENS-adjacent niches.

Human and translational studies support the plausibility of EGC involvement in motility-related dysfunction. Primary human EGCs exposed to inflammatory stimuli exhibit altered purinergic signaling, calcium responses, and mechanosensitivity, processes relevant to ENS communication and transit regulation (109). Histopathological analyses in STC and related motility disorders have report reductions in enteric glial populations accompanying neuronal alterations, suggesting that glial network changes may parallel altered motor states in human tissue (91, 110). However, these findings do not establish whether glial loss or reactivity is a primary driver, an amplifier, or a consequence of chronic dysmotility.

Preclinical models indicate that inflammatory or stress-related stimuli can induce enteric gliosis characterized by cytokine release, altered purinergic signaling, and immune recruitment (111). Interleukin-1 receptor type I (IL1R1) signaling has emerged as a regulator of glial activation; EGC-specific IL1R1 deficiency attenuates immune cell infiltration and partially restores motility in postoperative ileus models (112). Whether comparable signaling hierarchies operate in chronic FC/STC, and at what intensity or disease stage, remains unknown.

Reactive EGCs may participate in self-reinforcing neuroimmune loops. Extracellular ATP and P2X receptor signaling can promote MAPK activation and pro-inflammatory gene expression, indirectly shaping macrophage activation and cytokine profiles. In endotoxemia models, glial reactivity correlates with neuronal injury markers and suppressed peristalsis, whereas pharmacological or genetic attenuation of gliosis partially restores motility (111). These experimental observations suggest that glial reactivity can influence ENS function under inflammatory or endotoxemic conditions.

Despite these insights, direct evidence for a causal role of reactive gliosis in human FC/STC remains limited. Critical uncertainties include the triggers and thresholds of EGC reactivity, the distinction between adaptive and maladaptive glial responses, the spatial relationship between reactive glia and specific enteric neuronal subtypes, and the extent to which glial activation reflects upstream immune perturbation rather than a primary driver of ENS vulnerability (113).

Taken together, mast cell–nerve microdomains, macrophage plasticity, and reactive EGCs provide plausible cellular mechanisms by which ENS-adjacent neuroimmune signaling may influence neuromuscular function. However, because most evidence remains preclinical or extrapolated from adjacent conditions, the downstream Effector changes discussed below should be interpreted as potential consequences of Hub dysregulation rather than as inevitable outcomes of a confirmed cascade.

## 6 The effector: ENS vulnerability and motility failure

In the final component of the proposed Trigger–Gateway–Hub–Effector framework, the ENS is conceptualized as the downstream “Effector” through which microbiota-associated, epithelial, immune, and glial signals may converge to influence gastrointestinal motility. I Among the four components of the framework, downstream neuromuscular alterations have relatively stronger human histopathological support, particularly in severe or refractory STC. However, these findings should not be generalized to FC as a whole.

Importantly, available evidence does not imply homogeneous pathology across all FC phenotypes. Instead, human and experimental data suggest that advanced STC may occupy the severe end of a disease spectrum in which tissue-level neuromuscular alterations become more prominent than in normal-transit constipation or defecatory disorders. Reported features include reduced enteric neuronal density, neurochemical remodeling, and disruption of pacemaker-related ICC networks. However, these findings are inconsistent across studies, not universally present, and likely reflect late-stage or refractory disease subsets.

Conceptually, downstream ENS-related alterations can be organized into three partially overlapping domains: (i) neuronal loss or degenerative-like remodeling, (ii) compensatory or maladaptive plasticity involving neurochemical and phenotypic changes, and (iii) synaptic dysfunction accompanied by ICC network disruption. These processes should not be interpreted as a fixed sequence. Rather, they may interact dynamically and contribute to persistent hypomotility in selected STC phenotypes. This framework does not redefine FC as a structural neuromuscular disorder but highlights that ENS vulnerability may become more relevant in severe or treatment-resistant STC, with potential implications for therapeutic resistance.

### 6.1 Neuronal loss and degenerative remodeling

Neuronal loss and degenerative-like remodeling are downstream features reported mainly in subsets of severe or refractory STC and less consistently in broader FC populations. These changes may arise in association with chronic low-grade neuroimmune perturbation, oxidative stress, altered trophic support, or prolonged bowel stasis rather than overt inflammatory tissue damage. In experimental systems, sustained exposure to stress-related mediators, including cytokines, mast cell–derived proteases, and reactive oxygen species, can promote neuronal apoptosis, synaptic dysfunction, and network simplification. Whether comparable processes occur to the same extent in human FC/STC remains uncertain.

**Figure 3:**
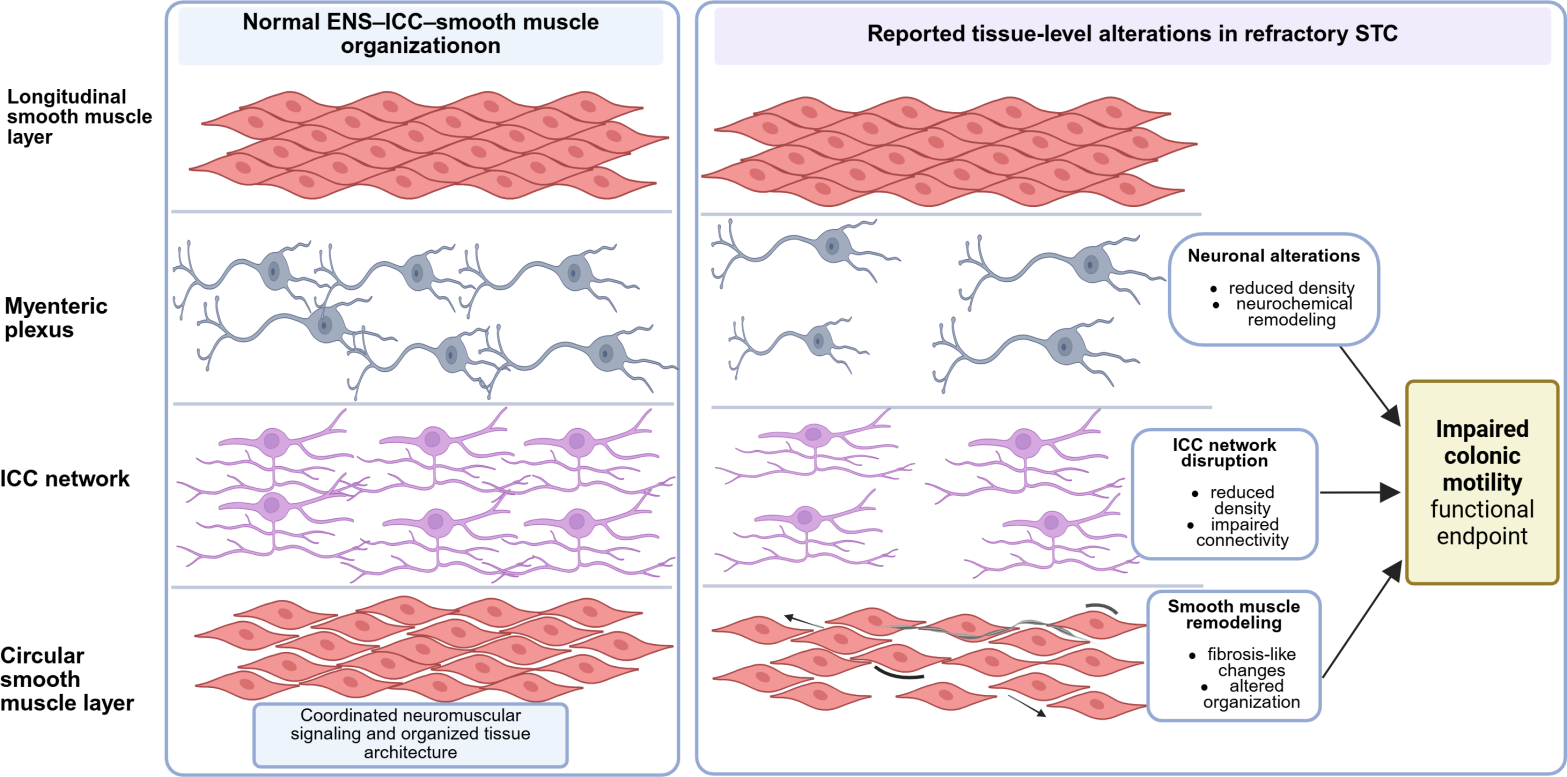
Effector-level tissue alterations reported in refractory slow-transit constipation. The schematic contrasts normal ENS–ICC–smooth muscle organization with tissue-level alterations reported mainly in severe or refractory STC. Under physiological conditions, myenteric neurons, interstitial cells of Cajal, and smooth muscle layers form an integrated neuromuscular unit that supports coordinated colonic motility. In refractory STC, reported alterations include enteric neuronal changes, ICC network disruption, and smooth muscle remodeling or fibrosis-like changes. These are presented as parallel tissue-level outcome nodes associated with impaired colonic motility, rather than as a confirmed sequential causal chain. Upstream immune–glial mechanisms that may contribute to these alterations remain incompletely validated in human FC/STC and are therefore not depicted as established causal pathways in this figure. These findings should not be generalized to all FC phenotypes. Created in BioRender. Ge, D. (2026) https://BioRender.com/see1q8u.

Reactive EGCs may further influence neuronal stress by altering trophic support, purinergic signaling, and mediator release, thereby contributing to local neuroimmune remodeling in experimental settings (89, 113). Human histopathological studies of STC have reported reductions in ICCs alongside diminished enteric neuronal structures across colonic regions, with some studies linking these alterations to delayed transit and impaired motor patterns (95, 114) (**Figure 3**). As summarized in **Figure 3**, these downstream findings are best interpreted as parallel tissue-level outcome nodes reported mainly in severe or refractory STC, rather than as a confirmed sequential causal chain. Alterations in specific neuronal subpopulations and neurotransmitter systems, particularly cholinergic and non-adrenergic, non-cholinergic pathways, have also been described, although direct and consistent quantification of inhibitory or nitrergic neuron loss in human STC remains limited (115, 116).

Local neuroimmune microenvironments, in which macrophages and MCs are positioned near neurons and glia, can release cytokines, growth factors, and bioactive mediators capable of influencing neuronal survival or functional state. Gut microbial dysbiosis and epithelial barrier vulnerability have been associated with chronic low-grade immune activation and increased exposure to microbial products in several gastrointestinal contexts, including conditions with disturbed ENS homeostasis (117, 118). However, in human FC/STC, direct evidence linking dysbiosis, barrier vulnerability, immune activation, and neuronal loss within the same patients is still lacking.

Despite the histopathological observations described above, mechanistic evidence in human FC/STC remains limited. Critical gaps include the temporal relationship between immune activation and neuronal loss, selective vulnerability of ENS neuronal subtypes, and the extent to which observed structural changes reflect primary injury, secondary adaptation to chronic stasis or distension, or both. Whether immune activation in STC acts as a causal driver, disease amplifier, or consequence of prolonged hypomotility remains unresolved.

### 6.2 Plasticity and phenotypic switching

When neuronal are stress or loss, the remaining enteric neurons may change their functional and phenotypic plasticity. These changes may include altered neurotransmitter expression, receptor profiles, synaptic connections, and neurotrophic signaling. Based on broader ENS biology, these changes may first help preserve remaining motility. But they may become harmful if they last too long, become too strong, or are poorly coordinated. These processes are well characterized in inflammatory and injury models. But their magnitude, persistence, and clinical relevance in human STC remain incompletely defined.

Plasticity in the adult ENS is regulated by intrinsic and extrinsic cues, including neurotrophic factors such as glial cell line–derived neurotrophic factor (GDNF) and brain-derived neurotrophic factor (BDNF)., These factors support neuronal survival and network adaptation beyond development (119, 120). Communication among enteric neurons, glial cells, and resident macrophages further shapes structural and functional plasticity under homeostatic and inflammatory conditions. Surviving neurons may display neurochemical remodeling. This may appear as changed proportions of cholinergic, nitrergic, and VIP-expressing neurons. These changes may shift the excitatory–inhibitory balance of motor circuits (63, 121). Structural plasticity, including axonal sprouting and partial reinnervation of denervated regions, has been observed in experimental models and may be guided by enteric glial signaling (122, 123).

Clinically, such plastic adaptations may transiently compensate for neuronal loss or impaired signaling. But maladaptive remodeling may worsen hypomotility in advanced disease. Examples include inhibitory signaling, impaired excitatory drive, or aberrant synaptic organization. Evidence for these processes in human FC/STC remains limited. The boundary between adaptive and maladaptive plasticity is also poorly defined. Clarifying these boundaries is essential for understanding whether ENS plasticity represents a protective response, a contributor to persistent dysfunction, or both.

### 6.3 Synaptic dysfunction and ICC network disruption

Synaptic dysfunction and ICC network disruption are nearby mechanisms that may contribute to persistent motility impairment in severe or refractory STC. ICCs act as pacemaker cells. They generate and spread slow-wave electrical activity, coordinate smooth muscle contractions, and help transmit signals between enteric neurons and smooth muscle (124).

In STC, histopathological studies often report reduced ICC density, particularly near the myenteric plexus and circular muscle layer, often accompanied by reductions in ENS neuronal structures (95, 114). These findings are summarized in **Figure 3** as Effector-level tissue alterations associated with impaired colonic motility, while their upstream immune–glial causes remain incompletely validated in human FC/STC.

Mechanistically, ICC disruption may reflect downstream consequences of immune-mediated stress, oxidative injury, altered neuronal input, or loss of trophic support from neurons and glia. ICC depend on c-Kit/stem cell factor signaling. So, inflammatory cytokines or reactive oxygen species may impair ICC viability or promote phenotypic conversion, as reported in other motility disorders (125). Experimental models of ICC deficiency show problems in excitatory cholinergic and inhibitory nitrergic neurotransmission. This highlights the importance of intact ICC networks in neuromuscular coupling (126, 127).

Clinically, correlations among ICC loss, slow-wave abnormalities, symptom severity, and transit delay in STC remain variable, and the causal relationship between immune alterations and ICC disruption in humans has not been fully established (128). It remains unclear whether ICC loss represents a primary pathogenic event, a secondary response to neuronal injury or chronic hypomotility, or an adaptive remodeling process in chronically distended bowel (129). Differential vulnerability among ICC subpopulations and the potential reversibility of ICC network disruption remain important unresolved questions (95, 129).

Integrating neuronal loss, phenotypic plasticity, synaptic dysfunction, and ICC network alterations within the proposed framework highlights how upstream microbiota–immune perturbations may converge on ENS effector mechanisms in selected STC phenotypes. However, this convergence should be interpreted as a plausible and testable model rather than evidence of a uniform structural pathology across all FC phenotypes.

These Effector-level alterations also help explain why some patients with severe or refractory STC may respond poorly to therapies that target stool consistency or acute motility alone. Nevertheless, because the causal relationships among dysbiosis, immune remodeling, ENS vulnerability, and ICC disruption remain uncertain, therapeutic strategies should be discussed according to evidence strength rather than assumed mechanism. The following section therefore considers microbiota-directed, immune-modulatory, neuroprotective, and multi-component interventions using an evidence-weighted translational framework.

## 7 Therapeutic implications: evidence-weighted translational perspectives

Within the proposed Trigger–Gateway–Hub–Effector framework, FC/STC is conceptualized as a heterogeneous condition in which microbiota-associated, epithelial, immune, glial, and neuromuscular processes may interact in selected patient subgroups. However, because direct causal links among these layers remain incompletely validated in humans, therapeutic implications should be interpreted according to the strength and source of available evidence.

Current standard therapies, including osmotic and stimulant laxatives, secretagogues, and 5-HT4 receptor agonists, primarily target stool hydration secretion, or motility. These treatments remain clinically important and should not be displaced by unvalidated mechanistic approaches. Rather, the framework discussed here may help identify future research directions for patients with persistent or refractory symptoms, particularly those with objectively documented delayed colonic transit.

Accordingly, therapeutic strategies are discussed below using an evidence-weighted structure that preserves the logic of upstream microbial modulation, Hub-level immune–glial regulation, downstream neuroprotective support, and multi-component intervention. These strategies are presented as symptom-supported, preclinical, or conceptual examples rather than established mechanism-based clinical recommendations.

### 7.1 Microbiota modulation: human symptom data with indirect mechanistic support

Gut microbiota modulation represents a clinically upstream strategy because dysbiosis has been associated with constipation-related symptoms, stool characteristics, and intestinal transit phenotypes (8). However, direct evidence linking microbiota-targeted interventions to the prevention of enteric neuronal or ICC injury, reactive gliosis, or ICC network disruption in human FC/STC remains limited. Most human studies evaluate symptom endpoints, such as bowel movement frequency, stool consistency, bloating, or quality of life, whereas mechanistic insights are derived largely from animal, ex vivo, or *in vitro* studies *in vitro* studies.

Probiotic, prebiotic, and synbiotic interventions may alter microbial composition and metabolic output, including enrichment of *Bifidobacterium* and *Lactobacillus* species and increased SCFA production. Clinical studies and meta-analyses in FC suggest modest and heterogeneous symptom improvement, primarily reflected in bowel frequency or stool consistency (130, 131). Experimental data suggest that SCFAs, particularly butyrate, may influence epithelial integrity and enteroendocrine signaling pathways relevant to motility regulation (130). However, translation to ENS-related or ICC-related outcomes in human FC/STC remains uncertain.

Beyond metabolic precursors, FFAR2 (GPR43) and FFAR3 (GPR41) have emerged as potential pharmacological targets for mimicking microbiota-derived signaling (132). These receptors are activated by SCFAs and are expressed in enteroendocrine and enteric neuronal cells, supporting their role in microbiota–host communication (133). Their activation has been associated with gut hormone secretion and neuronal modulation; however, direct regulation of serotonin (5-HT) release and cholinergic neuronal function in human FC/STC remains incompletely defined (134). Evidence for specific effects on cholinergic neuronal function is limited. Therefore, SCFA–FFAR signaling should currently be regarded as a plausible mechanistic target rather than a validated therapeutic pathway.

Fecal microbiota transplantation (FMT) has shown potential symptomatic benefit and increased microbial diversity in limited constipation cohorts (135, 136). Some clinical and experimental evidence suggests that FMT may reshape fecal bile acid profiles, including secondary bile acids, and may influence TGR5 (GPBAR1)-mediated signaling pathways relevant to motility (52). Mechanistically, bile acids can activate TGR5expressed on colonic enterochromaffin cells and intrinsic primary afferent neurons, leading to the release of 5-HT and calcitonin gene-related peptide (CGRP), which participate in initiation of the peristaltic reflex (49). Nevertheless, causal links among FMT, bile acid restoration, immune stabilization, ENS preservation, and ICC network integrity have not been established in human STC.

Overall, microbiota-directed interventions may modulate luminal metabolites and microbial-derived signals, thereby indirectly influencing epithelial barrier function and mucosal immune tone. These changes could plausibly affect processes associated with neuronal vulnerability, reactive gliosis, or ICC disruption described earlier in the framework, but direct human evidence remains lacking. Substantial uncertainties persist regarding microbial targets, donor selection, treatment duration, long-term safety, inter-individual variability, and ENS-related outcomes. Within the present framework, microbiota modulation is best positioned as an upstream symptom-modifying and stratification-relevant strategy rather than a standalone disease-modifying treatment.

### 7.2 Hub-level immunomodulation: mainly preclinical and adjacent-disease evidence

Hub-level immunomodulatory approaches are conceptual strategies aimed at stabilizing neuroimmune interactions involving MCs, macrophages, EGCs, and enteric neurons. Supporting evidence derives largely from preclinical studies and indirect observations in adjacent disorders of gut–brain interaction, particularly irritable bowel syndrome (IBS), rather than FC/STC-specific investigations (20). Therefore, these strategies should be interpreted as biologically plausible but clinically unvalidated in constipation.

Mast cell stabilizers, such as ketotifen and cromolyn, have shown symptom-modulating effects in IBS, whereas evidence specific to constipation-predominant phenotypes or STC remains limited (137, 138). These data support the general concept that mast cell–nerve signaling can influence gut sensorimotor function, but they do not establish that mast cell stabilization improves delayed transit or protects the ENS in FC/STC.

Targeted cytokine inhibition, including TNF-α or IL-1β-related pathways, has not been validated for ENS protection, ICC preservation, or motility improvement in FC/STC. Modulation of muscularis macrophage phenotypes through CSF1 receptor-related pathways has been explored experimentally, but clinical translation to constipation disorders is absent (106). Similarly, EGCs contribute to neuroinflammatory signaling in experimental models, and modulation of IL-1– or purinergic-related pathways can influences glial activation and motility outcomes in inflammatory or postoperative ileus models (111, 112). Whether comparable signaling hierarchies operate in chronic FC/STC remains unknown.

Within the proposed framework, immunomodulation is therefore framed as a theoretical intermediate strategy that may reduce low-grade immune activation and limit ENS-adjacent neuroimmune stress. Critical uncertainties remain regarding patient selection, timing, durability, safety, and inter-individual immune–microbiota heterogeneity. At present, this tier should be viewed as conceptual and preclinical rather than clinically established.

### 7.3 Effector-level neuroprotection: preclinical and conceptual evidence

No established neuroprotective therapies exist for FC or STC. Effector-level neuroprotection is discussed here as a conceptual strategy aimed at preserving enteric neuronal function, enteric glial support, and ICC network integrity in severe or refractory STC. However, the reversibility of advanced ENS or ICC alterations in chronic constipation remains uncertain. Experimental studies indicate that neurotrophic factors such as GDNF and BDNF support enteric neuronal survival and circuit plasticity (120, 139). Antioxidant and anti-apoptotic agents mitigate inflammatory and oxidative stress in enteric neurons, while SCF/c-Kit signaling is critical for ICC maintenance and pacemaker activity (140, 141). Attenuation of inflammatory mediators and reactive gliosis has also been associated with improved neuronal environments in preclinical models (142). These findings support biological plausibility but do not establish clinical efficacy in FC/STC.

Phytochemicals and bioactive compounds have also been investigated in experimental contexts. Neferine, a bioactive alkaloid, has been reported to protect EGCs from oxidative damage through activation of PINK1/Parkin-mediated mitophagy. This process has been associated with preservation of neurotrophic support, as indicated by increased levels of GDNF and nerve growth factor (NGF) in experimental STC contexts (143). However, such findings remain preclinical and should not be interpreted as evidence for validated neuroprotective therapy in human constipation.

From a translational perspective, combined modulation of neurotrophic support, oxidative stress, and local inflammatory tone may represent a theoretical approach to preserving ENS integrity, particularly in severe or refractory STC (144). Key gaps include optimal intervention timing, long-term safety, selection of appropriate biomarkers, and the extent to which neuronal or ICC dysfunction is reversible in chronic disease. Neuroprotection is therefore presented as a complementary research concept rather than a primary therapeutic target.

### 7.4 Multi-component interventions and mechanism-guided stratification: conceptual future directions

Multi-component interventions are presented as conceptual illustrations of how several layers of the proposed framework might be engaged simultaneously. This category includes dietary strategies, microbiota-directed combinations, host–microbe metabolic modulation, and selected traditional Chinese medicine formulations. However, these approaches should not be presented as validated mechanism-based therapies unless direct clinical and mechanistic evidence is available.

Certain Traditional Chinese Medicine formulations, such as MaZiRenWan, have demonstrated improvements in symptom-based endpoints in randomized trials. These outcomes primarily reflect bowel function and patient-reported symptoms and do not do not by themselves provide direct evidence of targeted immune modulation, or ENS preservation, or ICC restoration.

Proposed mechanistic insights derive largely from hypothesis-generating network pharmacology analyses and preclinical studies, suggesting possible associations between bioactive constituents and pathways related to motility, epithelial function, and inflammation (145). Animal experiments further suggest that some formulations alter gut microbial composition and metabolites, with correlations to motility changes (146). Much of this evidence originates from IBS, IBD, or experimental models, and extrapolation to FC or STC should be approached cautiously (147).

To date, no studies have directly demonstrated multi-component formulations modulate periganglionic immune niches or preservation of ENS integrity in human constipation disorders. Within the present framework, multi-target approaches are therefore presented as illustrative examples of systems-oriented therapeutic thinking rather than mechanistically validated treatment paradigms.

Future mechanism-guided studies should aim to identify patient subgroups in whom layered interventions are biologically plausible. A realistic near-term target may be refractory STC patients with objectively documented delayed colonic transit, because this subgroup is clinically identifiable and more likely to show tissue-level neuromuscular alterations. Additional candidate subgroups may include methane-positive constipation, inflammatory biomarker-positive constipation, and patients with combined microbiota and bile acid abnormalities.

Based on this framework, several testable hypotheses emerge: (i)whether immune cells are enriched near the myenteric plexus in subsets of refractory STC; (ii)whether fecal SCFA or bile acid profiles correlate with ICC density or objective transit delay; and (iii) whether enteric glial activation markers are associated with delayed colonic transit or treatment resistance. Systematic validation of these predictions in human cohorts is essential for refining and contextualizing this conceptual model.

A structured, evidence-weighted overview of representative therapeutic strategies, categorized by proposed framework node, evidence source, and interpretive strength, is provided in **Table 2**.

**Table 2:** Evidence-weighted overview of therapeutic strategies relevant to microbiota–immune–ENS interactions in FC/STC.

Strategy	Framework node	Main evidence source	Current interpretation
Probiotics/prebiotics/synbiotics	Trigger	Human FC symptom studies and preclinical mechanistic studies	Symptom-supported; ENS/ICC mechanisms unproven
FMT	Trigger	Limited human cohorts and animal/ex vivo studies	Early symptom data; disease-modifying effects unproven
Bile acid/TGR5 modulation	Trigger/Gateway	Human STC observations and animal models	Biologically plausible; therapeutic validation limited
SCFA–FFAR2/3 modulation	Trigger/Gateway	Mainly preclinical/*in vitro* studies	Mechanistically plausible; clinical role unclear
Mast cell stabilization	Hub	Human IBS data; limited FC/STC evidence	Adjacent DGBI evidence; not validated for STC
Macrophage/cytokine modulation	Hub	Animal, postoperative ileus, inflammatory models	Preclinical; no established clinical role in FC/STC
Enteric glial modulation	Hub/Effector	Preclinical inflammatory/endotoxemia models	Conceptual; human relevance unknown
Neurotrophic/antioxidant strategies	Effector	Animal and cellular studies	Preclinical only
Phytochemicals	Effector/multi-target	Animal and cellular STC	Hypothesis-generating
TCM formulations	Multi-layer/conceptual	Symptom-based trials and network pharmacology analyses	Symptom data for some; mechanisms unvalidated

## 8 Discussion and future perspectives

Current evidence supports an association between gut microbiota changes and FC or STC. But the overall evidence remains moderate. It mainly comes from human observational studies and experimental models, not direct human mechanistic data (31). Human observational studies have reported compositional and functional differences in the gut microbiota of patients with constipation (148). However, mechanistic links among dysbiosis, epithelial barrier vulnerability, microbial signal translocation, immune remodeling, and ENS dysfunction are still inferred. The main support comes from animal models, *in vitro* studies, or indirect disease contexts (53, 149).

Within the intestinal wall, immune remodeling has been reported in STC, including increased macrophage infiltration and immune-related transcriptional changes in colonic tissues, suggesting that immune alterations may accompany selected constipation phenotypes or disease stages (150). Conceptual frameworks proposing coordinated interactions among immune cells, enteric glia, and enteric neurons provide a useful structure for interpreting these findings. Nevertheless, direct spatial and functional validation of such neuroimmune microenvironments in human FC/STC remains limited, and their pathogenic relevance is not yet established. Much of the current understanding in this area is supported by animal studies or inferred from related gastrointestinal disorders, rather than direct human evidence.

In contrast, downstream neuromuscular alterations have relatively stronger human histopathological support, particularly in severe or refractory STC. Multiple histopathological studies in STC patients have demonstrated reduced density of ICC and structural changes within the ENS, findings that have been associated with delayed colonic transit (95, 140, 151). These observations provide clinical support for the relevance of neuromuscular remodeling in selected STC phenotypes. However, they do not establish the upstream causes of these alterations, and direct causal relationships and temporal sequencing among microbial, immune, and neural alterations remain incompletely defined.

From a translational standpoint, this integrative perspective supports an evidence-weighted therapeutic paradigm encompassing modulation of the gut microbiota, regulation of immune or glial responses, and preservation of neuromuscular function. Interventions targeting the microbiota, including dietary strategies, probiotics, synbiotics, and fecal microbiota transplantation, have demonstrated variable symptomatic benefits in chronic constipation (152). However, evidence for direct effects on ENS or ICC integrity in humans remains limited. Broader system-level therapeutic approaches, including multi-component or integrative strategies, may conceptually align with modulation across multiple pathological layers, but their mechanism-specific actions in FC/STC require clarification through well-designed translational and clinical studies.

The Trigger–Gateway–Hub–Effector framework proposed in this review may provide a conceptual basis for mechanism-based clinical stratification of FC/STC. However, this framework should not be interpreted as assigning fixed disease drivers to all patients. In some mild or moderate FC phenotypes, Trigger- or Gateway-level processes, such as microbiota dysbiosis, metabolite alterations, epithelial barrier vulnerability, or mucosal immune activation, may be more prominent and potentially more reversible. These patients may be more suitable for studies evaluating microbiota-directed, dietary, secretory, or barrier-supportive interventions (141, 153). In contrast, refractory STC with objectively documented delayed colonic transit may be the most realistic near-term target for mechanism-based stratification, because this subgroup is clinically identifiable and more likely to show tissue-level neuromuscular alterations, including ENS remodeling, ICC depletion, or muscularis immune changes (154).

Recent evidence indicates that patients with different constipation subtypes may exhibit distinct microbial compositions, supporting the view that STC, normal-transit constipation, and defecatory disorders are pathophysiologically heterogeneous and may require different assessment and treatment strategies (148). This layered model emphasizes the need for personalized evaluation based on dominant biological features rather than a one-size-fits-all approach. Candidate stratification markers may include objective colonic transit delay, methane positivity, fecal SCFA or bile acid profiles, inflammatory biomarkers, epithelial barrier markers, and tissue-level immune–ENS or ICC changes when biopsy or surgical specimens are available.

A central unresolved question is whether immune and ENS alterations in refractory STC are drivers, amplifiers, or consequences of prolonged stasis. At present, these possibilities cannot be clearly separated. Immune remodeling and ENS changes may have different roles at different disease stages. In some patients, they may act as early amplifiers. In others, they may result from long-term hypomotility or distension. In advanced disease, they may become part of self-sustaining feedback loops. Long-term stasis may also change microbial metabolism, epithelial stress, immune status, and neuromuscular signaling. This may create circular relationships, not only linear relationships.

A further significant challenge pertains to the determination of the admissibility of evidence pertaining to the proposed Gateway-to-Hub transition as direct human evidence. The most compelling evidence will require spatially resolved, patient-matched analyses that correlate epithelial barrier vulnerability, microbial products or metabolites, immune-cell localization, and changes in tissues adjacent to the ENS within the same FC/STC patient. Promising research approaches include paired mucosal–muscularis biopsies, multiplex immunofluorescence, spatial transcriptomics, single-cell RNA sequencing, microbial metabolite analysis, imaging of perineural barrier markers in the myenteric plexus, and correlation analysis with objective measures of colonic motility. Moreover, longitudinal sampling before and after targeted interventions will facilitate the establishment of the chronology of events and causal relationships. Several knowledge gaps remain. First, direct human evidence linking specific microbial metabolites or immune phenotypes to ENS alterations in FC/STC is scarce. Second, validated biomarkers that can group patients by main pathological features are lacking. Third, longitudinal studies assessing whether early interventions can modify long-term neuromuscular outcomes are limited. Fourth, most therapeutic studies use symptom endpoints and do not assess immune–ENS or ICC-related outcomes.

Future research should focus on human-centered and integrative approaches. These approaches should combine microbiota profiling, metabolomics, immune characterization, epithelial barrier assessment, ENS and ICC assessment, and objective measures of motility. Development of translational biomarkers that reflect immune–neural interactions and neuromuscular integrity may help group patients by mechanism. In this context, FC/STC can be viewed as conditions involving disrupted communication across microbial, epithelial, immune, glial, and neural systems.
